# PPAR*γ* Agonists as an Anti-Inflammatory Treatment Inhibiting Rotavirus Infection of Small Intestinal Villi

**DOI:** 10.1155/2016/4049373

**Published:** 2016-06-13

**Authors:** Dory Gómez, Natalia Muñoz, Rafael Guerrero, Orlando Acosta, Carlos A. Guerrero

**Affiliations:** Departamento de Ciencias Fisiológicas, Facultad de Medicina, Universidad Nacional de Colombia, Bogotá, Distrito Capital, Colombia

## Abstract

Rotavirus infection has been reported to induce an inflammatory response in the host cell accompanied by the increased expression or activation of some cellular molecules including ROS, NF-*κ*B, and COX-2. PPAR*γ* stimulation and N-acetylcysteine (NAC) treatment have been found to interfere with viral infections including rotavirus infection. Small intestinal villi isolated from* in vivo* infected mice with rotavirus ECwt were analyzed for the percentage of ECwt-infected cells, the presence of rotavirus antigens, and infectious virion yield following treatment with pioglitazone. Isolated villi were also infected* in vitro* and treated with PPAR*γ* agonists (PGZ, TZD, RGZ, DHA, and ALA),* all-trans* retinoic acid (ATRA), and NAC. After treatments, the expression of cellular proteins including PPAR*γ*, NF-*κ*B, PDI, Hsc70, and COX-2 was analyzed using immunochemistry, ELISA, immunofluorescence, and Western blotting. The results showed that rotavirus infection led to an increased accumulation of the cellular proteins studied and ROS. The virus infection-induced accumulation of the cellular proteins studied and ROS was reduced upon pioglitazone treatment, causing also a concomitant reduction of the infectious virion yield. We hypothesized that rotavirus infection is benefiting from the induction of a host cell proinflammatory response and that the interference of the inflammatory pathways involved leads to decreased infection.

## 1. Introduction

Rotavirus is the leading cause of severe acute dehydrating diarrhea in infants and young children worldwide. According to the available figures, this viral agent causes approximately 527,000 under-five deaths every year [1]. Rotavirus is member of the family Reoviridae and its nonenveloped, tripled-layered particle (TLP) contains a genome of 11 dsRNA segments encoding six structural proteins (VP1–VP4, VP6, and VP7) and six nonstructural proteins (NSP1–6) [2]. Rotaviruses induce inflammatory and oxidative stress signaling pathways in infected cells, while anti-inflammatory and antioxidant treatments have shown inhibiting rotavirus infection [3–5]. Recently, evidence has been provided that thiazolidinediones (TZDs), such as pioglitazone (PGZ) and rosiglitazone (RGZ), inhibit rotavirus infection in MA104 cultured cells [6] and ICR mice [3]. This inhibition has been suggested to occur through the thiazolidinedione-mediated activation of peroxisome proliferator-activated receptor-gamma (PPAR*γ*), a ligand-activated transcription factor belonging to the nuclear receptor gene family, which shows anti-inflammatory properties [7, 8]. Activation of PPAR*γ* by TZDs interferes with the NF-*κ*B signaling cascade, leading to a decrease in the transcription of some NF-*κ*B-dependent proinflammatory genes [9]. PPAR*γ* ligands inhibit the expression of inflammatory genes such as interleukin- (IL-) 1*β* and tumor necrosis factor- (TNF-) *α* [7]. PPAR*γ*, like PPAR*α* and PPAR*β*, is widely expressed in a broad range of cells in which it regulates the transcription of distinct genes through heterodimerization with the retinoid X receptors (RXRs) [10, 11].

PPAR*γ* activation has been associated with inhibition of some viral infections. Stimulation of PPAR*α* and PPAR*γ* was reported to block human immunodeficiency virus- (HIV-) 1 replication and tumor necrosis factor- (TNF-) *α* production in acutely infected cells [12]. Downregulation of the respiratory syncytial virus- (RSV-) induced ICAM-1 expression and nuclear factor NF-*κ*B activity has been observed after treatment of human lung epithelial cells with PPAR*γ* agonists [13]. RGZ inhibits the hepatitis B virus (HBV) replication in HepG2 cells transfected with a plasmid containing HBV genome [14]. NF-*κ*B constitutes a family of transcription factors which play essential roles in inflammation, immunity, cell proliferation, differentiation, and survival [15]. NF-*κ*B has been reported to mediate cyclooxygenase-2 (COX-2) expression [16] while inhibition of COX-2 activity has been associated with reduction of rotavirus infection [4]. NF-*κ*B activation has been observed in intestinal epithelial cells (IECs) following infection with rotaviruses [17, 18], although some rotavirus strains can inhibit the activation of NF-*κ*B [19].

Two rotavirus vaccines (Merck's RotaTeq and GlaxoSmithkline's Rotarix) have been introduced in more than 100 countries since May 2007. To date, there are 53 countries offering them through their national immunization programs [20]. The assessment of these vaccines has indicated that they are significantly effective in reducing the burden and aftermath of this viral disease [21]. Although vaccine therapy is still the best hope to protect children from rotavirus infection a number of factors are affecting the introduction of these vaccines into national immunization programs in developing countries [17]. Financial and logistic problems are challenging the potential of rotavirus vaccines for reducing the risk of death from diarrhea. The attenuated live nature of the current vaccines has raised some concerns related with viral shedding, risk of transmission [22], the potential lack of coverage for all serotypes [23], and a slightly elevated risk of intussusception [21, 24]. Alternate strategies for improving response to existing vaccines have been suggested to address the remaining rotavirus-associated burden of mortality and morbidity [17].

In the search of alternative antiviral strategies that may improve the treatment of diarrheal disease caused by rotavirus and contribute to the understanding of the mechanism underlying rotavirus infection cycle, we have assayed the potential of PGZ in the control of rotavirus infection. A thorough understanding of the mechanisms supporting rotavirus infection is vital for comprehending better the inhibitory effect based on PPAR*γ* agonists and antioxidants [5, 6, 25]. Drawing on the fact that PGZ and RGZ were able to inhibit rotavirus infection in mice [3], we wanted to extend our previous findings by testing additional PPAR*γ* agonists such as alpha-linolenic acid (ALA), 13-(S)-hydroxyoctadecadienoic acid (13(S)-HODE) (HODE), and docosahexaenoic acid (DHA) besides all-trans retinoic acid (ATRA) and the previously tested PPAR*γ* agonists PGZ and RGZ. In addition, we wanted to know whether the expression of some cellular proteins previously related with rotavirus infection is responsive to viral infection and treatment with some PPAR*γ* agonists using a synchronous system consisting of small intestinal villi isolated from mice. Here we show that the cellular proteins PPAR*γ*, NF-*κ*B, PDI, and Hsc70 and reactive oxygen species (ROS) were increased by* in vivo* and* in vitro* rotavirus infection of small intestinal villi, whereas their increased levels were returned close to basal ones, with concomitant reduction of viral infection, following treatment with PGZ and other PPAR*γ* agonists. It is proposed that rotavirus infection is sensitive to the expression of genes regulated by transcription factors binding to PPAR*γ* response elements (PPREs) and ATRA response elements (RAREs).

## 2. Materials and Methods

### 2.1. Animals, Virus, and Reagents

The wild-type murine rotavirus EDIM-Cambridge (ECwt) G3P [18] was a kind gift from Dr. M. Franco (Instituto de Genética, Pontificia Universidad Javeriana, Bogotá, Colombia). Fifty-two-day-old ICR mice weighing 25–30 g were obtained from the Instituto Nacional de Salud, Bogotá, Colombia. The animal experimentation protocol was duly approved by the Committee of Ethics of the School of Medicine according to the national and international regulations.

Hyperimmune antiserum against cesium chloride-purified ECwt was raised in rabbit, goat, mouse, and guinea pig. Rabbit hyperimmune antisera to NSP4, NSP5, PDI, and Hsc70 were obtained using the respective purified antigens. All preimmune sera were tested in Western blotting for the absence of antibodies against rotavirus. Goat anti-PDI (SC-17222), anti-Hsc70 (SC-1059), anti-PPAR*γ* (SC-6285). and anti-COX-2 (SC-18619) polyclonal antibodies (Abs) (200 *µ*g/mL) were purchased from Santa Cruz Biotechnology, Inc. (Santa Cruz, CA, USA). Rabbit anti-PPAR*γ* (SC-7273), anti-p-NF-*κ*B p50 (Ser 337) (SC-271908), anti-COX-2 (SC-19999), and anti-PDI (SC-376369) monoclonal antibodies (mAbs) (200 *µ*g/mL) were also from Santa Cruz Biotechnology, Inc. (Santa Cruz, CA, USA). Rabbit anti-NF-*κ*B p50 were obtained from Invitrogen (Carlsbad, CA, USA). Horseradish peroxidase- (HRP-) conjugated goat anti-rabbit IgGs (SC-2313), goat anti-mouse IgG-HRP (SC-2005), and HRP-conjugated rabbit anti-goat IgGs (SC-2020), fluorescein isothiocyanate- (FITC-) conjugated donkey anti-mouse IgGs (SC-2024), and FITC-conjugated goat anti-rabbit IgGs (SC-2359) (400 *µ*g/mL) were purchased from Santa Cruz Biotechnology, Inc. (Santa Cruz, CA, USA). Cy5-labeled goat anti-rabbit IgG Abs (SC-45101, 200 *µ*g/mL, Santa Cruz Biotechnology, Inc., Santa Cruz, CA, USA) and Alexa 594-labeled rabbit anti-goat IgG Abs (400 *µ*g/mL, Invitrogen, Carlsbad, CA, USA) were used. ROS were detected using the Cellomics Oxidative Stress 1 HCS Reagent Kit (Cat # 8401001, Thermo Scientific, Waltham, MA, USA).

Drugs used were according to the United States Pharmacopeia grade and consisted of active ingredients lacking excipients. Antioxidants, NAC and* all-trans* retinoic acid (ATRA) and alpha-linolenic acid (ALA), were purchased from MP Biomedicals (Solon, OH, USA). PPAR*γ* agonists thiazolidinedione (TZD), pioglitazone (PGZ), and rosiglitazone (RGZ) were provided by Alfa Aesar (Ward Hill, MA USA), Sigma (St Louis, MO, USA), and Santa Cruz Biotechnology Inc (Santa Cruz, CA, USA), respectively. Docosahexaenoic acid (DHA) was obtained from Santa Cruz Biotechnology Inc (Santa Cruz, CA, USA), and 13-(S)-hydroxyoctadecadienoic acid (13(S)-HODE) (HODE) was purchased from Amresco (Solon, OH, USA). Drugs were solubilized in the solvent indicated by manufacturers and then diluted in sterile phosphate buffered saline (PBS) and sterilized by filtration through 0.22 *μ*m membranes (Millipore, Bedford, MA, USA).

### 2.2. Intestinal Villi Isolation

Villi from small intestines were isolated as previously described [25]. The villus-enriched preparation in minimum essential medium (MEM) containing antibiotic/antimycotic solution was kept at 4°C until assayed for rotavirus infection, for no longer than 1 h.

### 2.3. Virus Infection Assay

Fifty-two-day-old male ICR mice were orally infected or not with purified ECwt as previously described [25]. 2 h after infection (h.p.i.) the mice were orally given PGZ (9 mg/kg/day) three times daily for two days. Control uninfected mice were given placebo (sterile PBS) or PGZ under the same dose conditions [3]. Following 12, 24, 36, and 48 h.p.i., mice were euthanized and their small intestines isolated. Duodenum samples were taken separately to be submitted for histopathological examination and the remaining intestines were used for villus isolation. The experiments here described were referred to as* in vivo* experiments.

Adult noninfected ICR mice were slaughtered and their small intestine separated for villus isolation. Villi were inoculated with rotavirus ECwt (MOI of 0.5) and after 15 min, PGZ (153 *µ*M), TZD (153 *µ*M), RGZ (153 *µ*M), DHA (45 *μ*M), ALA (45 *μ*M), HODE (45 *μ*M), or ATRA (45 *μ*M) was added separately and incubated at 37°C. The PGZ-treated villi were harvested every 2 h.p.i. whereas the villi treated with the other reagents were harvested at 12 h.p.i. Noninfected villi treated with PGZ (153 *µ*M) and harvested every 2 h.p.i. were used as a control. The experiments here described were referred to as* in vitro* experiments.

Villi isolated from* in vivo* infected mice were treated* in vitro* with PGZ (153 *µ*M) and cultured for 12 h at 37°C. Villi from* in vivo* infected and PGZ-treated mice were also cultured under the same conditions. Villi isolated from uninfected and PGZ-treated mice were also cultured for 12 h at 37°C. Villi isolated from uninfected mice were infected with ECwt (MOI of 0.5), treated or not with PGZ (153 *µ*M), and left for 12 h at 37°C. Villi isolated from uninfected mice were treated with PGZ (153 *µ*M) for 30 min at 37°C and then infected with ECwt (MOI of 0.5) and left for 12 h at 37°C. Villi were harvested after 12 h.p.i. at 37°C. In all cases, viral antigen and cellular proteins were analyzed by capture ELISA. The experiments here described were referred to as* in vivo-in vitro* experiments.

### 2.4. Viral Titers of Villus Lysates

Villi isolated from uninfected mice were seeded in 96-well culture plates and then infected or not with ECwt (MOI of 0.5). After 15 min p.i., PGZ (153 *µ*M), TZD (153 *µ*M), RGZ (153 *µ*M), DHA (45 *μ*M), ALA (45 *μ*M), HODE (45 *μ*M), or ATRA (45 *μ*M) was added to villi and left for incubation at 37°C for a maximum of 12 h. Control villi were incubated in the absence of reagents. After incubation, PGZ-treated villi were harvested every 2 h.p.i. whereas those treated with the remaining reagents were harvested every 12 h.p.i. Villi were subjected to two cycles of freeze and thaw and sonicated at 30% amplitude for 3 min with 30 sec sonication intervals. The villus lysates were added with 10 *µ*g/mL trypsin and serially diluted before inoculation to villi isolated from uninfected mice. Villi were incubated for 12 h at 37°C prior to immunochemistry analysis for rotavirus antigen-positive cells as indicated below. Results were graphed as FFU/mL versus postinoculation time.

### 2.5. Immunocytochemistry

Intestinal villi from infected and uninfected mice that had been treated or not with PGZ were fixed for 20 min in ice-cold methanol : acetic acid (3 : 1 v : v). After permeabilization in 0.5% Triton X-100 and 0.1% SDS for 15 min, the villi were incubated with 50 mM NH_4_Cl for 30 min. After washing with PBS, the villi that adhered to coverslips were incubated at 37°C with primary rabbit Abs against rotavirus structural proteins or primary rabbit Abs against rotavirus nonstructural proteins (NSP4 or NSP5). After washing with PBS, the villi were incubated with HRP-conjugated goat anti-rabbit Abs for 1 h at 37°C. The reaction was visualized using the aminoethylcarbazole (AEC) substrate. The villi were observed microscopically at magnification ×100 (VanGuard).

The percentage of cells being positive to viral antigen was estimated for villi isolated from infected and noninfected mice. The drug effect on virus infection was established by comparing the mean percentages of virus-infected villus cells from virus-infected mice treated with PGZ with those from virus-infected mice without PGZ treatment. The drug effect was expressed as mean percentage of rotavirus antigen-positive cells relative to that of infected mice without PGZ treatment. The same procedure was used for the analysis of villi infected* in vitro* that had been treated or not with PGZ, TZD, RGZ, DHA, ALA, HODE, and ATRA.

The effect of PPAR*γ* activation by PGZ, TZD, RGZ, DHA, ALA and HODE, or ATRA treatment on ECwt infection and expression of cellular proteins NF-*κ*B (phosphorylated and nonphosphorylated), PPAR*γ*, PDI, Hsc70, or COX-2 was analyzed by immunofluorescence using the above-prepared coverslips that had been used for the immunocytochemistry analysis of rotavirus antigen. These coverslips were washed with PBS and treated with primary goat Abs against PDI or Hsc70, rabbit Abs against p-NF-*κ*B p50 (Ser 337) and NF-*κ*B, or mouse mAbs against PPAR*γ* and incubated for 1 h at 37°C. Secondary FITC-conjugated donkey anti-goat, anti-rabbit, or anti-mouse Abs were applied for 20 min at room temperature (RT). Cells of villi were observed in a fluorescence microscope (VanGuard) and photographed.

Histological cross sections of mouse duodenum were also analyzed for the expression of the above-mentioned cellular proteins (NF-*κ*B, PPAR*γ*, PDI, Hsc70, and COX-2) and rotavirus infection. Duodenal cross sections from infected and uninfected mice that had been treated or not with PGZ were fixed with 10% paraformaldehyde, embedded in paraffin, and sectioned into 3–5 *µ*m thick slices. Paraffin-embedded sections were placed onto coverslips, deparaffinized in xylene, and permeabilized with 1% Triton X-100 and 1% SDS for 30 min. Following washing with PBS, the coverslips were treated with 50 mM NH_4_Cl for 30 min and then incubated with rabbit Abs to rotavirus structural proteins for 1 h at 37°C. Secondary HRP-conjugated goat anti-rabbit Abs were added and incubated for 1 h at 37°C. The reaction was visualized with AEC chromogen. The coverslips were reacted with goat anti-PDI, anti-Hsc70, anti-COX-2, or anti-PPAR*γ* Abs or rabbit anti-NF-*κ*B Abs for 1 h at 37°C. FITC-conjugated donkey anti-mouse Abs or FITC-conjugated donkey anti-rabbit Abs were added and incubated for 20 min at RT in the darkness. Representative photographs from each coverslip were taken using a fluorescence microscope (VanGuard). The analysis of photographs from immunocytochemistry and immunofluorescence analyses was conducted using the program ImageJ. The corrected total cell fluorescence (CTCF) values were converted into percentage values and represented as histograms. Values of uninfected controls were normalized to 100%.

Confocal microscopy was used for the analysis of cellular protein expression. Coverslips with deparaffinized sections were simultaneously incubated with goat Abs against p-NF-*κ*B p50 (Ser 337), NF-*κ*B and PDI, mouse mAbs against PPAR*γ*, and mouse, rabbit, or goat Abs against rotavirus structural proteins. After incubation for 1 h at 37°C, the coverslips were incubated for 20 min at RT with secondary FITC-conjugated goat anti-rabbit Abs or FITC-conjugated donkey anti-mouse Abs, Cy5-labeled goat anti-rabbit IgG Abs, or Alexa 594-labeled rabbit anti-goat IgG Abs. Confocal analysis was conducted to examine coverslips containing villi from infected and uninfected mice that had been treated or not with PGZ. The coverslips were observed, photographed using a confocal microscope (Nikon C-1), and analyzed using the colocalization finder plug-in of ImageJ.

### 2.6. ELISA

Villi isolated from mice under different experimental conditions were lysed in RIPA buffer (50 mM Tris-HCl, pH 8.0, 150 mM NaCl, 1% NP-40, 0.1% SDS, 0.5% DOC, and 0.5 mM PMSF) and sonicated at 30% amplitude for 3 min with 30 sec sonication intervals. ELISA plates were coated with guinea pig Abs to rotavirus structural proteins, goat Abs to PPAR*γ*, NF-*κ*B, COX-2 or rabbit Abs to PDI, or Hsc70 and blocked with 5% skimmed milk. Villus lysates from mice used in the* in vivo* experiments were applied at a total protein concentration of about 80 *µ*g/mL whereas those from the* in vitro* experiments were applied at about 20 mg/mL concentration. Villus lysates from uninfected mice or PBS instead of villus lysates were used as a control. Rabbit Abs against rotavirus structural proteins were added to well plates that had been coated with guinea Abs to rotavirus structural proteins. For detection of cellular proteins, goat Abs against PDI or Hsc70 and mouse mAbs against PPAR*γ*, NF-kB, or COX-2 were added to the well plates coated with the respective capture antibodies to the cellular proteins tested. HRP-conjugated donkey anti-goat Abs or HRP-conjugated goat anti-mouse Abs and o-phenylenediamine dichloride (OPD) (Thermo scientific 34005, Waltham, MA, USA) were used to visualize and quantify the reaction.

### 2.7. ROS Detection

ROS formation was measured using the Cellomics Oxidative Stress 1 HCS Reagent Kit. Villi from infected and uninfected mice that had been treated or not with PGZ were collected at the indicated postinfection times and placed in Dulbecco's modified Eagle media (DMEM), added with dihydroethidium (DHE) (16 *μ*g/mL final concentration) and Hoechst 33342 (0.016 ng/mL final concentration) and incubated for 30 min at 37°C. The villi were collected by centrifugation at 400 g for 2 min and then treated with 4% paraformaldehyde in PBS for 30 min at 37°C. The villi were placed onto slides which were embedded with resin and covered with coverslips. ECwt-infected and PGZ-treated villi* in vitro* were subjected to the same procedures. Ten representative image fields per slide were microscopically recorded.

### 2.8. Flow Cytometry

Flow cytometry analysis of NF-*κ*B, NF-*κ*B-p, PDI, Hsc70, PPAR*γ*, or COX-2 expression in villus cells isolated from infected or uninfected mice that had been treated or not with PGZ was carried out by fixing the PBS/EDTA- (5 mM) isolated villus cells in methanol : acetic acid (3 : 1 v/v) for 1 h at 4°C. The villi were incubated with 50 mM NH_4_Cl for 30 min and then treated with a mix containing primary Abs against the rotavirus structural proteins and cellular proteins NF-*κ*B, NF-*κ*B-p50, PDI, Hsc70, PPAR*γ*, and COX-2. Primary Abs were treated with secondary FITC-labeled donkey anti-mouse Abs and CY5-labeled goat anti-rabbit Abs. Cells without antibody treatment, cells with primary unrelated Ab treatment and secondary Ab treatment, and cells with secondary Ab treatment without primary Ab treatment were used as a control. The cells were analyzed in a flow cytometer (BD FACSCanto II).

### 2.9. Western Blotting

PPAR*γ* and NF-*κ*B-p50 expression in villus cells corresponding to the* in vitro* experiments was assessed by preparing villus lysates as indicated for ELISA. ECwt infection of villi was carried out in the presence or absence of PGZ (153 *µ*M), TZD (153 *µ*M), RGZ (153 *µ*M), DHA (45 *μ*M), ALA (45 *μ*M), HODE (45 *μ*M), or ATRA (45 *μ*M). The quantified lysates were treated with Laemmli's sample buffer and analyzed in 10% SDS-PAGE. The separated proteins were transferred to polyvinylidene fluoride (PVDF) membranes which were blocked with 5% skimmed milk and probed with primary goat anti-PPAR*γ* or rabbit anti-NF-*κ*B-p50 Abs. Secondary HRP-conjugated goat anti-rabbit or donkey anti-goat Abs were used to detect the reaction with the ImmunoCruz*™* Western blot luminol reagent kit (sc-2048 Santa Cruz Biotechnology, Santa Cruz, CA, USA).

### 2.10. Cell Viability

The viability of villus-associated cells was assessed using the trypan blue (Sigma 93595, St Louis, MO, USA) exclusion test. Villus suspensions from infected or uninfected mice that had been treated or not with PGZ, TZD, RGZ, DHA, ALA, HODE, or ATRA were tested. Cells excluding the stain were counted in 10 representative fields and their mean percentage referred to the total cells recorded.

### 2.11. Statistical Analysis

Statistical analyses of data were conducted using GraphPad Prism version 5.0 and applying the Kolmogorov-Smirnov test to determine whether sample data are normally distributed. Additionally, the parametric one-way analysis of variance (ANOVA) and multiple comparison Dunnett's test were used to find statistically significant differences.

## 3. Results

### 3.1. PPAR*γ* Activation Decreases ECwt Infection

**Figure 1:**
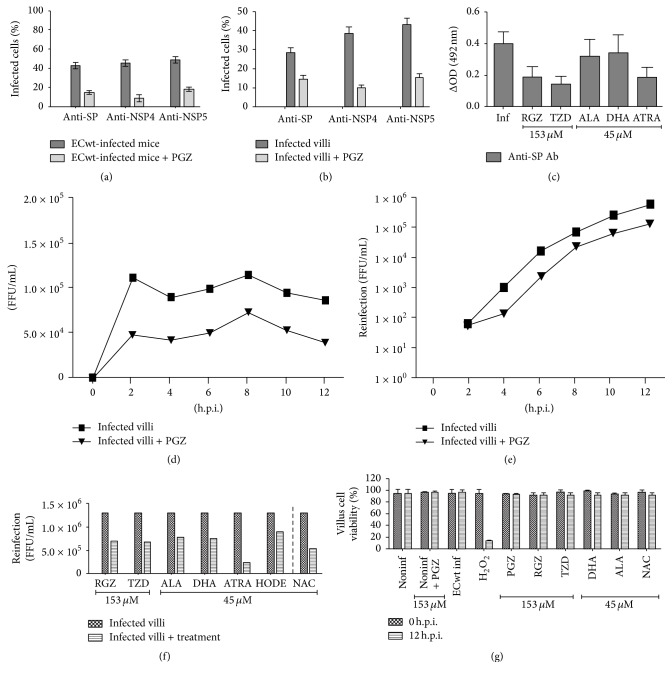
Effect of PPAR*γ* agonists and ATRA on ECwt infectivity in small intestinal villi. (a) Immunocytochemistry analysis using antibodies against viral structural proteins (SP) and nonstructural proteins (NSP4 and NSP5) present in villi from* in vivo* ECwt-infected mice (*n* = 3 per experiment) that had been treated or not with PGZ. (b) Immunocytochemistry analysis as in (a), except that villi isolated from mice (*n* = 3 per experiment) were infected* in vitro* with ECwt and treated or not with PGZ. (c) Capture ELISA analysis of lysates from* in vitro* ECwt-infected villi that had been treated or not with PGZ, RGZ, TZD, ALA, DHA, or ATRA. Viral antigen was detected with anti-SP antibodies (Ab). (d) Infectious titers of lysates from* in vitro* ECwt-infected villi that had been treated with PGZ. Viral titers were determined by immunochemistry assay using anti-SP antibodies. (e) Infectious titers of lysates from* in vitro* ECwt-infected villi that had previously been inoculated with the villus lysates analyzed in (d) and treated again with PGZ. (f) Infectious titers of lysates from* in vitro* ECwt-infected villi that had previously been inoculated with lysates from villi treated with RGZ, TZD, ALA, DHA, or ATRA and then treated with these same PPAR*γ* agonists. (g) Cell viability of villus cells determined with the trypan blue exclusion test. H_2_O_2_ was used as a control. Data are presented as mean ± SD from three independent experiments performed in duplicate.

Evidence has been provided that infection of cultured cells by rotavirus can be inhibited by NAC, pioglitazone, or rosiglitazone treatment [3, 5]. We assessed the ability of PGZ to interfere with ECwt infection of small intestine villus-associated cells from orally infected mice. The immunocytochemistry results showed that the percentage of infected cells of villi from infected mice decreased following the PGZ treatment as compared with the percentage of infected cells of villi isolated from untreated control mice (Figure 1(a)). Following the immunocytochemistry analysis, the mean percentage of PGZ inhibition of viral infection through the 12–48 h.p.i. period tested for the presence of structural, NSP4 and NSP5 viral antigen was 65.09%, 80.52%, and 62.47%, respectively, as compared to the mean percentage of infection for the ECwt-infected villi without PGZ treatment (Figure 1(a)). Uninfected cells treated or not with PGZ did not react with the antibodies used against rotavirus structural and nonstructural proteins. These results show that PGZ treatment of ECwt-infected mice reduced significantly the viral infection.

The mean percentage of villus cells remaining positive to viral antigen after PGZ, TZD, RGZ, DHA, ALA, or ATRA treatment was also studied in a synchronous system consisting of small intestinal villi isolated from uninfected mice. The mean percentage of PGZ inhibition of viral infection through the 12–48 h.p.i. periods was determined in terms of the presence of ECwt structural and nonstructural (NSP4 and NSP5) antigens. The mean percentages of positive cells for these antigens were 48.81%, 73.96%, and 64.41%, respectively, when compared to the ECwt-infected control villi without PGZ treatment (Figure 1(b)). Cells present in uninfected villi were completely negative for viral structural and nonstructural antigens, irrespective of whether villi were treated or not with PGZ. These results show that the treatment of* in vitro* ECwt-infected villi with PGZ also caused a significant reduction in the number of villus cells accumulating the virus-encoded proteins tested. A similar behavior to that observed for PGZ-treated ECwt-infected villi was found for ECwt-infected villi after treatment with the other PPAR*γ* agonists tested and with ATRA as the total content of viral structural proteins accumulated was decreased. The ELISA OD values found for villus lysates from ECwt-infected villi treated with RGZ, TZD, ALA, DHA, or ATRA were 0.191 ± 0.064, 0.145 ± 0.048, 0.322 ± 0.107, 0.342 ± 0.114, and 0.186 ± 0.062, respectively, in comparison to 0.399 ± 0.079 for untreated ECwt-infected control villi (Figure 1(c)). No reaction for antibodies against viral structural proteins was detected in extracts from noninfected villi that have been treated or not with the indicated anti-inflammatory agents.

### 3.2. Effect of PGZ Treatment on the Titer of Infectious Particles

Although a decrease in the ECwt antigen was observed after PGZ treatment in both* in vivo* and* in vitro* systems, we wanted to study whether this antigen decrease was accompanied by an effective decrease in the proportion of infectious particles. The immunocytochemistry results of* in vitro* infected villi indicated that PPAR*γ* activation by PGZ treatment decreased the infectious titer by 57.37% (2 h), 53.49% (4 h), 49.70% (6 h), 36.36% (8 h), 44.34% (10 h), and 54.33% (12 h) through the period examined (from 2 to 12 h.p.i.) as compared with that of infected villi without pioglitazone treatment (Figure 1(d)). Infectious titer was completely absent in extracts from uninfected and uninfected plus PGZ control villi. The mean decrease in infectious titer caused by PGZ treatment was 49.28% through this postinfection time. To test for the effect of a second cycle of PGZ treatment on infectious titer, villi that had been infected and treated with pioglitazone were collected again every 2 h (from 2–12 h.p.i.), lysed, inoculated into uninfected villi in the absence of pioglitazone treatment, and incubated for 12 h. The immunochemistry results indicated that inocula from PGZ-treated villi showed significantly less infectious titer at the end of the 12 h incubation period in comparison with inocula from PGZ-untreated villi. The decrease in infectious titer was about 16.67% (2 h), 86.36% (4 h), 85.63% (6 h), 25.31% (8 h), 75.17% (10 h), and 78.28% (12 h) (Figure 1(e)), whereas the mean decreased was 61.23% through the postinfection period examined. Extracts from uninfected villi that had been treated (or not) with PGZ lacked infectious titers. These results suggest that the decrease in viral antigen observed in PGZ-treated villi was paralleled by a decrease in the number of infectious virions. The viral titer in crude lysates from ECwt-infected villi was reduced by an average of 46.15%, 47.69%, 40.77%, 42.31%, 82.31%, 31.54%, and 59.23% by treatment with RGZ, TZD, ALA, DHA, ATRA, HODE, and N-acetylcysteine (NAC), respectively, through the postinfection period tested (2–12 h.p.i.) (Figure 1(f)). Lysates from uninfected villi lacking infectious titer were used as a control. NAC treatment of ECwt-infected villi was used as a positive control of inhibition of rotavirus infection. The reagent concentrations used did not affect cell viability through the 12 h.p.i. period examined (Figure 1(g)). Overall, the results suggest that PPAR*γ* activation led to a decreased number of the infectious virions that are formed during the viral cycle.

### 3.3. PPAR*γ* Activation Modifies Expression of Cellular Proteins and Decreases ECwt Infection

**Figure 2:**
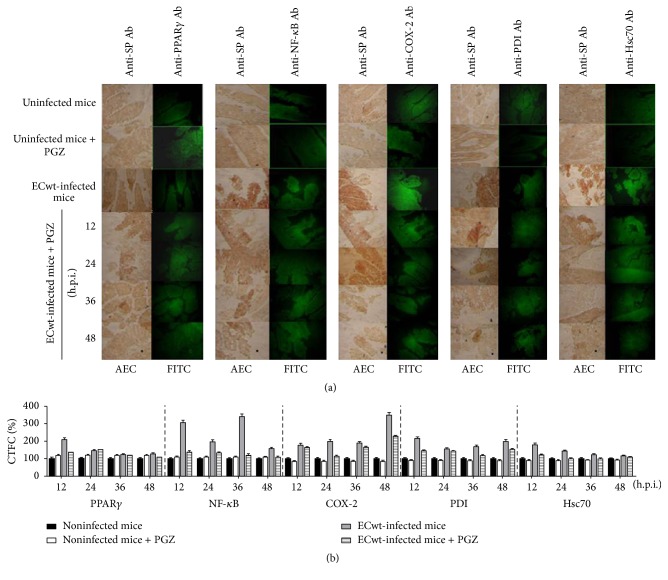
Expression of cellular proteins in villus cells from* in vivo* ECwt-infected mice treated with PGZ. (a) Mice (*n* = 3 per experiment) were infected or not with ECwt and then treated or not with PGZ. Villi isolated from mice were treated for immunochemistry analysis using primary antibodies against rotavirus structural proteins (SP) and secondary HRP-conjugated antibodies and AEC as a substrate. The same slides were subjected to immunofluorescence analysis using the indicated primary antibodies (Ab) and secondary FITC-conjugated antibodies. Representative micrographs are shown. (b) Fluorescence of cells positive to the cellular proteins indicated was measured in randomly chosen triplicate fields per slide using the ImageJ program, and the corrected total cell fluorescence (CTCF) values converted into percentage values are shown as mean (± SD) from three independent experiments performed in duplicate.

To characterize the influence of ECwt infection on the expression of cellular proteins PPAR*γ*, NF-*κ*B, COX-2, PDI, and Hsc70, an immunofluorescence assay was conducted using both the* in vivo* and* in vitro* systems in the presence or absence of treatment with PGZ. Similarly, an immunofluorescence assay was conducted using the* in vitro* systems in the presence or absence of treatment with RGZ, TZD, ALA, DHA, HODE, or ATRA. Representative photographs taken at the indicated time points after infection were analyzed using the program ImageJ (Figure 2(a)). These photographs suggest that viral infection increased the accumulation of the cellular proteins indicated, whereas PGZ treatment caused a decrease of the signals corresponding to these proteins and the viral antigens.

The fluorescence staining intensity was quantified using the program ImageJ. The corrected total cell fluorescence (CTCF) values indicated that* in vivo* treatment of uninfected mice with PGZ led to a mean increase of 20.34 ± 5.09% in PPAR*γ* expression through the 12–48 h.p.i. periods, when compared with its expression in villi from uninfected control mice (Figure 2(b)). ECwt infection of mice increased PPAR*γ* expression by 110.35 ± 10.5%, 46.02 ± 6.78%, 23.00 ± 4.80%, and 29.07 ± 5.39% in villus cells at 12, 24, 36, and 48 h.p.i., respectively, relative to uninfected control mice. PGZ treatment of infected mice caused a significant decline of virus-induced expression of PPAR*γ* only at 12 and 48 h.p.i. (35.56 ± 5.96% and 16.23 ± 4.03%, resp.), whereas this expression was kept almost unchanged after PGZ treatment at the intermediate postinfection times examined (24 and 36 h.p.i.) (Figure 2(b)). A slight decrease in expression level for cellular proteins COX-2, PDI, and Hsc70 was observed in villus cells from uninfected mice that had been treated with PGZ, except for NF-*κ*B expression that was slightly increased (Figure 2(b)). Virus infection induced an average increase of 152.23 ± 87,90%, 129.82 ± 81.01%, 83.79 ± 27.15%, and 40.86 ± 29.02% for NF-*κ*B, COX-2, PDI, and Hsc70 expression, respectively, relative to villi from uninfected control mice during the 12–48 h.p.i. period examined. A comparison of the expression level for NF-*κ*B, COX-2, PDI, and Hsc70 over the time-course infection showed that PGZ treatment of infected mice caused a significant reduction of their expression at all the postinfection times analyzed (12–48 h.p.i.). The PGZ-induced average reduction was 45.78 ± 16.78%, 23.93 ± 17.63%, 23.47 ± 10.90%, and 21.74 ± 12.11% for NF-*κ*B, COX-2, PDI, and Hsc70, respectively, through the 12–48 h.p.i. period examined (Figure 2(b)).

**Figure 3:**
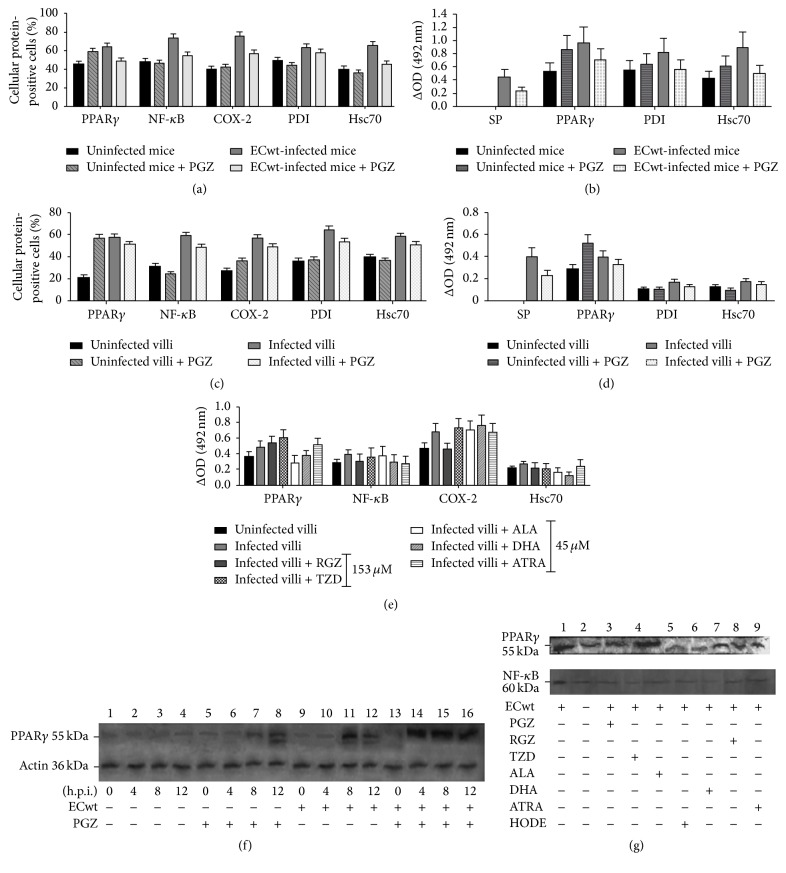
Expression of cellular proteins in villus cells from* in vivo* and* in vitro* ECwt-infected villi after treatment with PGZ. (a) Villi isolated from ECwt-infected mice (*n* = 3 per experiment) treated or not with PGZ were processed for immunofluorescence analysis using the primary antibodies against the indicated cellular proteins. Data are presented as mean percentage of villus cells being positive to the indicated proteins. (b) Villi isolated from ECwt-infected mice (*n* = 3 per experiment) treated or not with PGZ during the period 12 to 48 h.p.i. were lysed and analyzed by capture ELISA using detecting antibodies against the indicated proteins. Data are shown as mean values through the indicated period. (c) Villi isolated from mice were infected* in vitro* with ECwt and then treated or not with PGZ before being subjected to immunofluorescence analysis as indicated in (a). (d) Villi isolated from mice were infected* in vitro* with ECwt and treated or not with PGZ before being analyzed by ELISA as indicated in (b). (e) Villi isolated from mice were infected* in vitro* with ECwt, treated or not as indicated in (e), and analyzed by ELISA as indicated in (b). Results are presented as mean ± SD from three independent experiments performed in duplicate. (f) Villi isolated from mice were infected or not* in vitro* with ECwt and treated or not with PGZ before Western blotting analysis of villus lysates for the expression of PPAR*γ*. (g) Villi isolated from mice were infected or not* in vitro* with ECwt and treated or not with RGZ, TZD, ALA, DHA, and ATRA before Western blotting analysis using anti-PPAR*γ* and anti-NF-*κ*B antibodies.

The mean percentage of the immunofluorescent villus cells being positive to the cellular proteins PPAR*γ*, NF-*κ*B, COX-2, PDI, and Hsc70 in the* in vivo* system was 45.7 ± 3.38%, 48.4 ± 3.47%, 40.3 ± 3.17%, 49.6 ± 3.52%, and 40.3 ± 3.17%, respectively, for villi from uninfected mice, while the mean percentage of villus cells from ECwt-infected mice showing a positive signal for these proteins was 64.57 ± 4.02%, 74.15 ± 4.30%, 75.82 ± 4.35%, 63.65 ± 3.98%, and 65.85 ± 4.06%, respectively, through the time points examined (Figure 3(a)). These results are compatible with an increase of about 41.29%, 53.20%, 88.14%, 28.33%, and 63.4%, respectively, for these rotavirus-induced cellular proteins in comparison to the control villi from uninfected mice. In contrast, PGZ treatment of ECwt-infected mice produced a decrease in the mean percentage of villus cells expressing these cellular proteins. The mean percentage of PPAR*γ*, NF-*κ*B, COX-2, PDI, and Hsc70-expressing cells was reduced by 23.93%, 25.83%, 24.16%, 9.00%, and 30.57%, respectively, by PGZ treatment of infected mice through the 12–48 h.p.i. period when compared with the mean percentage of villus cells being positive to the corresponding cellular proteins tested in the villi from ECwt-infected mice without PGZ treatment (Figure 3(a)). According to these results, PGZ treatment of ECwt-infected mice appears to reduce the proportion of cells being positive to the cellular protein examined just to proportions that are similar to those observed in villus cells from uninfected mice.

The concomitant effect of ECwt infection and PGZ treatment on the expression level of the cellular proteins PPAR*γ*, PDI, and Hsc70 was examined. According to the ELISA results, ECwt infection significantly increased the expression level of PPAR*γ*, PDI, and Hsc70 from infected cells* in vivo*, leading to the following OD values: 0.966 ± 0.241, 0.832 ± 0.208, and 0.909 ± 0.227, respectively, through the postinfection time examined as compared with expression levels observed in uninfected control cells, which were 0.535 ± 0.133, 0.561 ± 0.140, and 0.435 ± 0.108 (Figure 3(b)). On the other hand, PGZ treatment of ECwt-infected mice* in vivo* reduced the viral structural antigen through the 12–48 h.p.i. period studied. The mean ELISA OD values for PPAR*γ*, PDI, and Hsc70 from pioglitazone-treated ECwt-infected mice were 0.711 ± 0.177, 0.571 ± 0.142, and 0.505 ± 0.126, respectively (Figure 3(b)). No viral structural protein was detected in villi from both uninfected and uninfected PGZ-treated control mice. These ELISA values appear to be intermediate between those found for ECwt-infected and noninfected mice. However, the decreasing effect of PGZ on the PPAR*γ* expression level in the infected cells was nonsignificant in the last postinfection times examined, probably due to the fact that PGZ induced PPAR*γ* expression levels in villus cells from uninfected mice.

The immunofluorescence analysis of the villus-associated cells from PGZ-treated villi* in vitro* showed that the percentage of cells expressing the cellular proteins PPAR*γ*, NF-*κ*B, COX-2, PDI, and Hsc70 was 21.8 ± 1.76%, 31.7 ± 2.12%, 27.5 ± 1.98%, 36.5 + 2.28%, and 39.8 ± 2.38% (Figure 3(c)). After ECwt infection of villi* in vitro*, the percentage of cells recorded as positive for these cellular proteins was increased in all the cases as compared with that of the cells from uninfected control villi (Figure 3(c)). The mean percentage of positive cells for PPAR*γ*, NF-*κ*B, COX-2, PDI, and Hsc70 from ECwt-infected villi was 57.98 ± 2.88%, 59.07 ± 2.90%, 57.03 ± 2.85%, 64.68 ± 3.03%, and 58.24 ± 2.88%, respectively, throughout the time points examined. These mean percentages were reduced by 11.85%, 17.72%, 14.26%, 17.05%, and 12.5%, respectively, when ECwt-infected villi were treated with PGZ during the 0–12 h.p.i. periods (Figure 3(c)). However, PGZ treatment of uninfected villi led to an increased level of PPAR*γ* expression that was similar to that induced by ECwt infection (Figure 3(c)).

The ELISA results of the* in vitro* experiments showed that PGZ treatment of ECwt-infected villus reduced the viral antigen, when averaged through the 12–48 h.p.i. period. PGZ treatment of ECwt-infected villi reduced viral antigen by 46.66% relative to infected villi without treatment (Figure 3(d)). Similarly, PGZ treatment of ECwt-infected villi decreased expression level of PPAR*γ*, PDI, and Hsc70 by 26.40%, 31.37%, and 44.44%, respectively, when compared with infected villi that were not treated with PGZ (Figure 3(d)). Expression level of cellular proteins in PGZ-treated ECwt-infected villi was closely similar to that observed in uninfected control cells (Figure 3(d)). In contrast, PGZ treatment of uninfected villi increased PPAR*γ* expression level by 61.87%, whereas PDI and Hsc70 expression levels remained the same, relative to the uninfected and untreated control villi (Figure 3(d)). Both uninfected and uninfected PGZ-treated control villi were negative for viral structural antigen.

Treatment of ECwt-infected villi with RGZ, TZD, ALA, DHA, HODE, or ATRA and their effects on the expression level of the cellular proteins PPAR*γ*, NF-*κ*B, COX-2, and Hsc70 were also examined in the* in vitro* system using villi taken at the postinfection times indicated above for the immunochemistry assay. The immunofluorescence analysis of the villus-associated cells studied in the* in vitro* system showed that ECwt infection led to a significantly increased percentage of villus cells showing positive signals for the cellular proteins PPAR*γ*, NF-*κ*B, COX-2, and Hsc70 at 12 h.p.i., in comparison to that found in cells from uninfected control villi. Treatment of ECwt-infected villi with RGZ, DHA, and HODE did not cause any statistically significant effect on the proportion of PPAR*γ*-positive cells. Except ATRA and NAC treatments that had a significant stimulating effect on the proportion of PPAR*γ*-positive cells the remaining treatments kept this proportion essentially unchanged. The percentage of villus cells being positive to NF-*κ*B was only reduced significantly by treatments with ALA and ATRA, whereas the treatment with the remaining PPAR*γ* agonists kept this percentage almost unchanged. The percentage of COX-2-positive villus cells was reduced, but not significantly, by RZG, TZD, ALA, and ATRA treatments, whereas this percentage remained statistically unchanged after treatment with any of the remaining PPAR*γ* agonists tested. Except TZD, DHA, HODE, and NAC treatments, the remaining treatments led to a significantly reduced percentage of villus cells being positive to signals for Hsc70. These results show that the cellular proteins examined are differentially responsive to the indicated treatments. These findings call for further research aimed at understanding the molecular mechanisms underlying these differential responses.

The ELISA results showed that ECwt infection significantly led to an increased expression of PPAR*γ*, NF-*κ*B, COX-2, and Hsc70 at 12 h.p.i. as compared with the respective expression levels observed in uninfected control cells (Figure 3(e)). However, a differential effect of treatments with PPAR*γ* agonists and ATRA on the cellular protein expression was observed for the ECwt-infected villi* in vitro*. PPAR*γ* expression was significantly reduced only by ALA treatment whereas the other treatments had no statistically significant effect on PPAR*γ* antigen accumulation in ECwt-infected villi (Figure 3(e)). In the case of NF-*κ*B expression, any of the reductions caused by the treatments assayed was statistically significant (Figure 3(e)). COX-2 expression was only reduced significantly by RZG treatment, whereas the remaining treatments keep its expression at levels that were statistically similar to that of the ECwt-infected villi without treatment (Figure 3(e)). Meanwhile, Hsc70 expression was reduced by all PPAR*γ* agonist treatments and ATRA treatment, except that DHA reduced significantly Hsc70 expression to levels even below the basal ones observed in the uninfected control villi (Figure 3(e)). Some of the differences observed in the effects caused by PPAR*γ* agonists and ATRA treatments could be explained by the fact that detection of the percentage of cells being positive for cellular proteins by immunofluorescence is less sensitive than detection by ELISA of the cellular antigen accumulated in the whole cell population.

Regarding that PGZ effects are PPAR*γ*-mediated, a Western blotting analysis was conducted for this host cell-encoded protein following ECwt infection of villi in an* in vitro* system. The infected villi were immediately treated or left untreated with PGZ, RGZ, TZD, ALA, DHA, HODE, or ATRA and then lysed at the indicated time points before Western blotting analysis using anti-PPAR*γ* or anti-NF-*κ*B antibodies. The results showed that PGZ treatment induced a higher expression of PPAR*γ* in noninfected villi at 8 and 12 h after treatment (Figure 3(f), lanes 7 and 8) as compared with the PGZ-untreated uninfected control villi at the same times (Figure 3(f), lanes 3 and 4). Basal expression levels of PPAR*γ* at 0, 4, 8, and 12 h after culture are shown in lanes 1, 2, 3, and 4, respectively (Figure 3(f)). The ECwt infection of PGZ-untreated villi led to further increased expression of PPAR*γ* at 8 h.p.i. (Figure 3(f), lane 11), whereas the expression of this cellular protein underwent a slight decrease at 12 h.p.i. (Figure 3(f), lane 12) in comparison with the immediately preceding time point examined. PGZ treatment of the ECwt-infected villi showed increased expression of PPAR*γ* at 4 (lane 14) and 8 h.p.i. (lane 15) followed by a slight decrease at 12 h.p.i. (lane 16) (Figure 3(f)). These findings suggest that pioglitazone treatment of ECwt-infected villi induces an additional expression of PPAR*γ* at least at the intermediate postinfection time examined. whether the effects of both PGZ treatment and ECwt infection are additive at least at the postinfection time examined remains to be studied. The ECwt-induced expression of PPAR*γ* was differentially affected by the treatment of villi with PGZ, RGZ, TZD, DHA, ALA, HODE, or ATRA when the analysis was made at 12 h.p.i. Whereas PGZ treatment, used as reference, appears to keep the PPAR*γ* expression nearly unchanged, TDZ treatment stimulated further the expression of this cellular protein (Figure 3(g)). In contrast, ALA and HODE produced a significant decrease of the PPAR*γ* expression level, whereas RGZ, DHA, and ATRA induced a slight decrease of the PPAR*γ* expression level when compared to the expression level found in ECwt-infected villi without treatments (Figure 3(g)). In the case of NF-*κ*B, its expression level was also increased by ECwt infection, whereas all of the treatments of infected villi with PGZ, RGZ, TZD, DHA, ALA, HODE, or ATRA appear to reduce the NF-*κ*B expression to levels similar to that found in uninfected control villus cells (Figure 3(g)).

To study the relationship between viral infection and cellular protein expression, a flow cytometry analysis of PPAR*γ*, p-NF-*κ*B, NF-*κ*B, COX-2, PDI, and Hsc70 was conducted. A significant reduction of the percentage of cells being positive to viral antigens was found in villus cells from infected mice that had been treated with PGZ as compared with the pioglitazone untreated control. For instance, the percentage of viral antigen-positive villus cells was decreased by 68.2% at 36 h.p.i., whereas this percentage was almost abolished at 48 h.p.i. in the case of PGZ-treated mice. Respecting cellular proteins, the percentage of villus cells from infected mice showing positive signals for the proteins assessed was significantly increased in comparison to that observed in control villus cells from uninfected mice. However, except at 36 h.p.i., PGZ treatment led to a decreased percentage of cells showing positive signals for these proteins. For all cellular proteins tested, this percentage declined approaching the basal levels observed in control villus cells from uninfected mice (Table 1).

**Figure 4:**
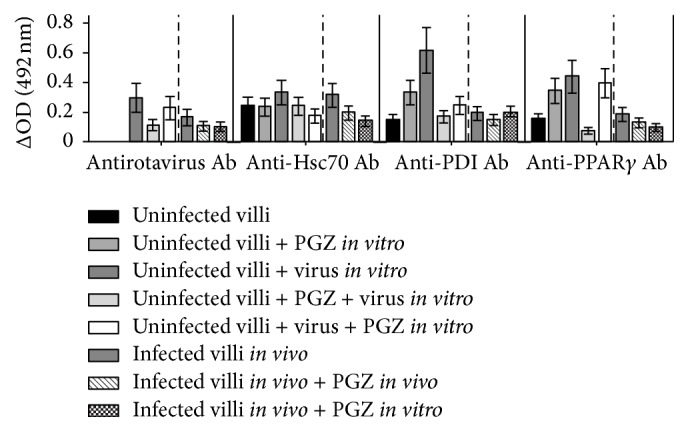
Effect of* in vitro* PGZ treatment on the expression of cellular proteins in villi isolated from* in vivo* ECwt-infected mice. Villi were isolated from ECwt-infected and noninfected mice (*n* = 3 per experiment) and then treated or not* in vitro* with PGZ. Villi were lysed and analyzed by ELISA using antibodies against rotavirus structural proteins (SP) and the indicated cellular proteins. Data are presented as mean ± SD from three independent experiments performed in duplicate.

To study the sensitivity of* in vivo* ECwt infection to PGZ treatment* in vitro*, small intestinal villi were isolated from infected mice at 48 h.p.i. and then treated* in vitro* with PGZ for 12 h. PGZ treatment effect on rotavirus SP and on cellular proteins PPAR*γ*, PDI, and Hsc70 was also determined using ELISA. The* in vitro* PGZ treatment of* in vivo* infected villi resulted in decreased levels of viral SP, PPAR*γ*, and Hsc70 by 37.19%, 48.40%, and 55.21%, respectively, whereas PDI level remained the same, in comparison to the respective antigens originally present in the control villi isolated from* in vivo* infected mice (Figure 4). The* in vitro* accumulation of viral SP, PPAR*γ*, PDI, and Hsc70 in villi isolated from infected mice that had been treated with pioglitazone* in vivo* increased by 35.97%, 31.38%, 23.71%, and 37.85%, respectively, in comparison to the respective antigens originally present in the control villi isolated from* in vivo* infected mice. These results confirm that small intestinal villi isolated from* in vivo* ECwt-infected mice continue to be able to support viral infection after isolation and* in vitro* culture. In addition, the results show that* in vitro* PGZ treatment of villi previously infected* in vivo* is efficient in interfering with rotavirus infection (Figure 4).

**Figure 5:**
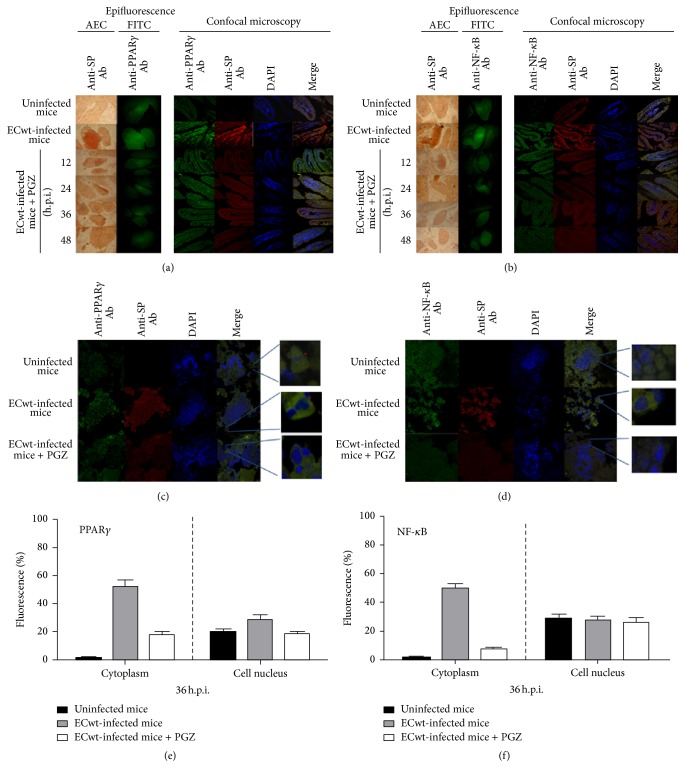
Immunochemistry, immunofluorescence, and confocal microscopy analyses of PPAR*γ* and NF-*κ*B in villus cells from ECwt-infected mice treated with PGZ. (a) Histological cross sections of duodenal villi from ECwt-infected mice that had been treated with PGZ were analyzed by immunocytochemistry for the presence of ECwt SP (EAC) or epifluorescence for the presence of PPAR*γ* (FITC). Histological cross sections were observed using confocal microscopy for the identification of PPAR*γ* (green) and rotavirus SP (red). Nuclei were counterstained with DAPI (blue). Representative photographs are shown. (b) Histological cross sections analyzed for the presence of NF-*κ*B and rotavirus SP as indicated in (a). (c) Villi isolated from ECwt-infected and noninfected mice that have been treated or not with PGZ were processed and analyzed at 36 h.p.i. for the subcellular location of PPAR*γ* and ECwt SP using confocal microscopy. Representative micrographs are shown. (d) Villi isolated from ECwt-infected or noninfected mice were treated or not as described in (c), except that they were analyzed for the subcellular location of NF-*κ*B and ECwt SP. Representative micrographs are shown. (e) Villi from ECwt-infected or noninfected mice (*n* = 3 per experiment) that had been treated or not with PGZ were subjected to confocal analysis for the quantification of subcellular location (nucleus or cytoplasm) of PPAR*γ*. (f) Villi from ECwt-infected or noninfected mice were treated as described in (e), except that they were analyzed for the quantification of subcellular location (nucleus or cytoplasm) of NF-*κ*B. Data are shown as mean ± SD from three independent experiments performed in duplicate.

To investigate the subcellular localization of viral antigens and cellular proteins studied in this work, a colocalization analysis by confocal fluorescence microscopy and epifluorescence was conducted. The analysis of histological cross sections of duodenum from orally ECwt-infected mice showed that PGZ treatment led to decreased immunofluorescence signals for both viral antigens and cellular proteins PPAR*γ*, NF-*κ*B, COX-2, PDI, and Hsc70 with respect to those from infected mice without pioglitazone treatment. A representative image of the PGZ treatment effect on the expression of cellular proteins is shown for PPAR*γ* and NF-*κ*B (Figures 5(a) and 5(b)). Quantification of colocalization of viral and cellular proteins using the colocalization finder plug-in in ImageJ yielded Pearson's correlation coefficients which were compatible with a random colocalization for these proteins. The percentages of villus cells from duodenum exhibiting PPAR*γ* or NF-*κ*B staining in the cytoplasm or nucleus were quantified at 36 h.p.i. using confocal microscopy analysis of histological cross sections of duodenal villi from ECwt-infected and uninfected mice that had been treated or not with PGZ (Figures 5(c) and 5(d)). PPAR*γ* immunofluorescence was detected in the cytoplasm of 1.5 ± 0.55%, 53.0 ± 3.26%, and 18.4 ± 1.92% of villus cell from uninfected, infected, and infected and PGZ-treated mice, respectively. The corresponding percentages for nuclear PPAR*γ* fluorescence were 20.2 ± 2.01%, 29.5 ± 2.43%, and 18.1 ± 1.90% (Figure 5(e)). These results suggest that ECwt infection dramatically increased the accumulation of cytoplasmic PPAR*γ* and, to a lesser extent, the accumulation of the nuclear PPAR*γ*. The percentages for NF-*κ*B fluorescence detected in the cytoplasm of villus cells from uninfected, infected, and infected and pioglitazone-treated mice were 1.7 ± 0.59%, 49.7 ± 3.15%, and 7.7 ± 1.24%, respectively, whereas the corresponding percentages for the nuclear NF-*κ*B fluorescence were 29.5 ± 2.43%, 28.2 ± 2.37%, and 27.0 ± 2.33% (Figure 5(f)). These percentages are indicating that the nuclear NF-*κ*B levels were practically insensitive to ECwt infection while the cytoplasmic NF-*κ*B levels were impressively increased. Taking into account that the* in vivo* infection system analyzed is an asynchronous one, the subcellular localization percentages obtained for PPAR*γ* and NF-*κ*B are reflecting an average state of the infection process rather than a synchronous viral cycle. However, the results suggest that ECwt infection increases the percentage of villus cells being positive to both cytoplasmic and nuclear PPAR*γ* in comparison to that of uninfected cells, whereas PGZ treatment appears to counteract this enhancing effect. The same trend was observed for NF-*κ*B, except that nuclear accumulation of NF-*κ*B seems to be unchanged by both ECwt infection and PGZ treatment.

### 3.4. Rotavirus Infection and Oxidative Stress

**Figure 6:**
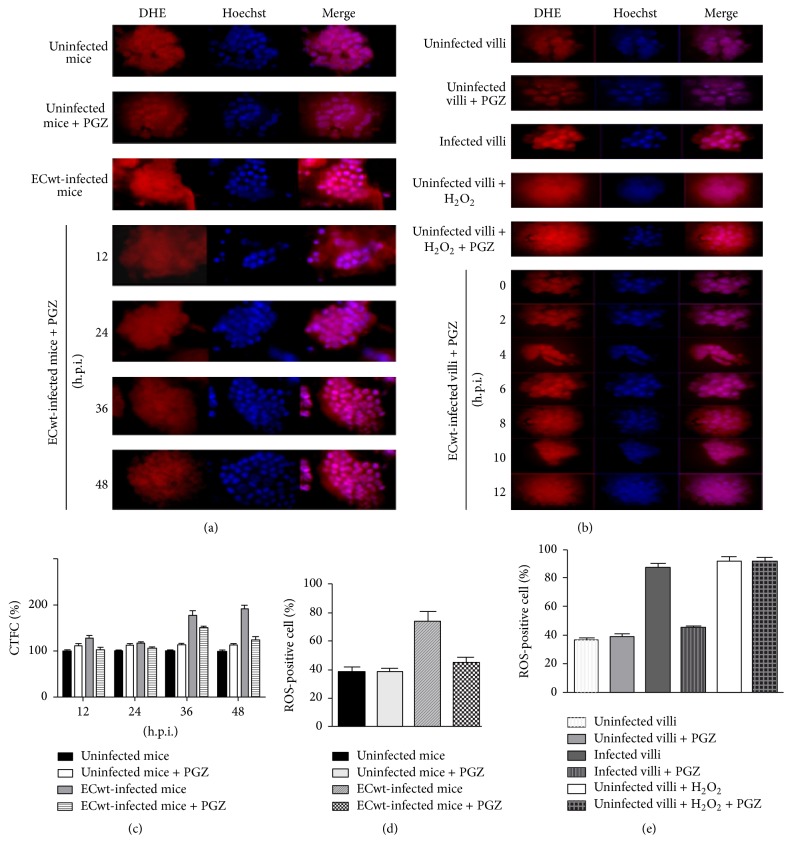
Oxidative stress induction by ECwt infection of small intestinal villi. (a) Small intestinal villi were isolated from EC-infected mice (*n* = 3 per experiment) that had been treated or not with PGZ. The isolated villi were tested for the presence ROS at the indicated postinfection times using Cellomics® Oxidative Stress 1 HCS Reagent Kit. Quantitative detection of ROS was performed with DHE. Villus cells were costained with dihydroethidium (DHE) and Hoechst for quantitative detection of ROS and nuclei staining, respectively. Representative micrographs are shown. (b) Small intestinal villi isolated from mice were infected with ECwt and treated or not with PGZ. Villi were collected at the postinfection times indicated and subjected to the procedures indicated in (a). Representative micrographs are shown. (c) Fluorescence of ROS-positive cells at the indicated postinfection times was measured in randomly chosen triplicate fields per slide using the ImageJ program, and the corrected total cell fluorescence (CTCF) values are shown in terms of mean (SD) percentages of uninfected control mice. (d) Small intestinal villi were isolated from EC-infected mice that had been treated or not with PGZ. Villus cells were processed as indicated in (a) and the ROS-positive cells recorded using a fluorescence microscope (VanGuard). Data are expressed as mean percentage of ROS-positive cells through the 48 h.p.i. period. (e) Small intestinal villi isolated from mice were infected* in vitro* with ECwt and treated or not with PGZ. Villus cells were processed as indicated in (a) and the ROS-positive cells recorded as indicated in (d). Data are shown as mean ± SD percentage of ROS-positive cells through the 12 h.p.i. period. Villi treated with H_2_O_2_ were used as a control. Data are from three independent experiments performed in duplicate.

An altered oxidative/antioxidative profile has been reported for homogenates of whole small intestine from rotavirus-infected neonatal mice [26]. In this context, we attempted to study whether rotavirus infection was able to induce oxidative stress in ECwt-infected cells of villi isolated from mouse small intestine. Villi isolated from infected mice that had been treated or left untreated with PGZ were compared with villi from uninfected mice in terms of the accumulated ROS. Data showed that, in average, 38.47 ± 3.50% of villus cells from the uninfected control mice were ROS-positive through the 48 h postinfection time period examined, whereas this mean percentage was increased to 74.29 ± 6.75% in the case of ECwt-infected villi. PGZ treatment of infected mice reduced the mean percentage of ROS-positive villus cells to 44.89 ± 4.08% (Figures 6(a) and 6(d)). In the experiments involving* in vitro* ECwt infection of villi isolated from uninfected mice, it was found that, in average, 87.66 ± 2.62% of villus cells were positive to ROS for the ECwt-infected villi in comparison to 36.57 ± 1.35% for uninfected control villi through a 12 h postinfection time course (Figure 6(b)). The mean percentage of ROS-positive cells for ECwt-infected villi was reduced to 44.92 ± 1.63% when these villi were maintained in the presence of PGZ (Figures 6(b) and 6(e)). The corrected total cell fluorescence (CTCF) values for ROS accumulated in villus cells showed that these species remained essentially unchanged through the postinfection times studied in the cases of uninfected and PGZ-treated uninfected mice (Figure 6(c)). Virus infection increased accumulation of ROS by 28.94 ± 5.38%, 16.28 ± 4.03%, 78.43 ± 8.86%, and 91.16 ± 9.55% at 12, 24, 36, and 48 h.p.i. in comparison with villi from uninfected mice. In contrast, PGZ treatment induced a decrease of ROS accumulated in villus cells from virus-infected mice. The percentage of this PGZ-induced decrease was 19.35 ± 4.39%, 8.46 ± 2.91%, 15.67 ± 3.95%, and 34.09 ± 5.83% at 12, 24, 36, and 48 h.p.i., respectively (Figure 6(c)). Overall, both* in vivo* and* in vitro* experimental results support the hypothesis that PGZ inhibits the ECwt-induced oxidative stress in small intestine villus cells.

## 4. Discussion

In the current paper, we study the* in vivo* and* in vitro* effect of PGZ and other PPAR*γ* agonist on rotavirus infection of small intestinal villus cells. Based on previous reports providing evidence on rotavirus infection inhibition by PGZ and RGZ [6, 25], we attempted to characterize some cellular and molecular events associated with ECwt infection of small intestinal villi using an* in vitro* synchronous system consisting of small intestinal villi isolated from mice instead of the* in vivo* asynchronous system represented by mice. The results from orally infected mice with ECwt showed that PGZ treatment reduced significantly the proportion of villus cells being positive to rotavirus antigens. This suggests that PGZ is able to affect viral infection by either direct contact with intestinal villus cells or returning from the systemic circulation back into the intestine. Whether PGZ affected* in vivo* rotavirus infection by reducing viral spreading or replication rate or both remains to be established. However, the results obtained using the mouse asynchronous system are reflecting average measurements from many viral life cycles at different stages. Interestingly, the PGZ inhibitory effect was confirmed in a synchronous* in vitro* system in which small intestine villi isolated from noninfected mice were infected with ECwt and then treated with PGZ at effective controlled concentrations. This treatment caused a significant reduction in the number of villus cells accumulating the virus-encoded proteins tested and also reduced the total amount of viral antigen present in lysates from the isolated villi. A similar behavior was found for ECwt-infected villi after treatment with other PPAR*γ* agonists such as RGZ, TZD, ALA, and DHA and also with ATRA, a physiologically active vitamin A metabolite that functions as a ligand for both the retinoic acid receptor (RAR) and the retinoid X receptor (RXR). Growth and differentiation of both normal and malignant cells are regulated by binding of the RAR-RXR heterodimer to retinoic acid (RA) response elements (RAREs) [27]. These findings suggest that activation of PPAR*γ* and ATRA-activated transcription factors is involved in the mechanism of rotavirus infection cycle.

Although a decrease of both the proportion of villus cells infected with ECwt and the total antigen accumulated in them was observed after PGZ treatment in both* in vivo* and* in vitro* systems, we wanted to study whether this inhibitory effect was accompanied by an effective decrease in the proportion of infectious particles. The immunochemistry results showed that inocula from PGZ-treated villi showed significantly less infectious titer at the end of the 12 h incubation period, in comparison to the pioglitazone untreated control. These results suggest that the decrease in viral antigen observed in PGZ-treated villi was paralleled by a decreased number of infectious virions. These results do not rule out that PGZ also affected rotavirus spreading as a reduced number of infected cells were also observed after PGZ treatment. Similar effect on infectious titer was found after treatment with other PPAR*γ* agonists such as RGZ, TZD, ALA, HODE, and DHA and also with* ATRA* and the antioxidant NAC.

In characterizing the influence of ECwt infection on the expression of cellular proteins PPAR*γ*, NF-*κ*B, COX-2, PDI, and Hsc70, we found that these cellular proteins were certainly increased in their expression following ECwt infection. ECwt-induced expression of these cellular proteins took place in terms of the increase of both the number of cells being positive to these proteins and the total amount of the protein antigen accumulated in villi. These results suggest that the progression of rotavirus infection seems to require the participation of these cellular proteins, without ruling out the implication of other cellular factors, different from those examined here. However, except for PPAR*γ*, which was only slightly reduced, the expression level of the cellular proteins studied returned closely to basal levels after PGZ treatment of ECwt-infected villi, suggesting that the decreased viral infection caused by PGZ treatment is involving at least the presence of PPAR*γ*, NF-*κ*B, COX-2, PDI, and Hsc70. However, the findings suggest that separately PGZ treatment and ECwt infection have a similar effect on PPAR*γ* expression in cultured villi. In addition, the effects of both PGZ treatment and ECwt infection seemed to be additive only in the intermediate postinfection times (4 to 8 h.p.i.) assayed in the* in vitro* system. In the case of the results obtained from the small intestinal villi isolated from* in vivo* ECwt-infected mice, these villi were able to continue supporting viral infection when they were further cultured in* in vitro* conditions. Interestingly, PGZ treatment of uninfected villi induced the PPAR*γ* expression. Moreover, PGZ treatment of these previously ECwt-infected villi was able to interfere with viral infection, suggesting that both* in vivo* and* in vitro* systems produced comparable results. Meanwhile, the immunofluorescence analysis showed that ECwt infection increased the percentage of villus cells being positive to both cytoplasmic and nuclear PPAR*γ*, whereas PGZ treatment appears to counteract this enhancing effect. The same trend was observed for NF-*κ*B, except that nuclear accumulation of NF-*κ*B seems to be insensitive to both ECwt infection and pioglitazone treatment. Although a differential effect of the PPAR*γ* agonists tested and ATRA on the expression of cellular proteins PPAR*γ*, NF-*κ*B, COX-2, and Hsc70 in ECwt-infected villi was observed, only PPAR*γ* expression was significantly reduced by ALA whereas COX-2 antigen was reduced only by RGZ treatment and Hsc70 expression was reduced by all PPAR*γ* agonist treatments and ATRA treatment. PPAR*γ* ligands such as EPA and DHA have been reported to increase PPAR*γ* expression and inhibit NF-*κ*B in dendritic cells [28].

Activation of PPAR*γ* by thiazolidinediones (TZDs) has been shown to interfere with the NF-*κ*B signaling cascade [9], which has been reported to mediate COX-2 expression [15] while inhibition of COX-2 activity reduces rotavirus infection [4]. NF-*κ*B activation has been observed in intestinal epithelial cells (IECs) following infection with rotaviruses [17, 18], although some rotavirus strains can inhibit the activation of NF-*κ*B [19]. PPAR*γ* activation has been shown to block TNF-*α* production [12], while NAC suppresses TNF-induced NF-*κ*B activation [28, 29] and inhibits rotavirus infection [3, 6]. On the other hand, rotavirus infection stimulates the expression of type I IFN and numerous cytokines that are part of the innate response of infected cells [18]. In addition, expression of many cytokines is dependent on the activation of the transcription factor NF-*κ*B that occurs once viral components are detected within the host cell. NF-*κ*B is important for the induction of IFN-*λ* and IFN-*β*. Some rotaviruses have evolved strategies for limiting NF-*κ*B activation [30] and avoiding the effects of IFN, at least during the early stages of infection [31]. ATRA treatment has been found to decrease expression of NF-*κ*B in human breast cancer cells (MCF-7) [32], although ATRA has been found to induces NF-*κ*B in normal human keratinocytes [33]. According to the frame of this rationale and our results, we hypothesize that the early rotavirus infection is benefiting from the induction of a host cell proinflammatory response as evidenced by the increased expression of some cellular molecules such as ROS and COX-2 and the increased expression and activation of NF-*κ*B. Interference of the proinflammatory process with PPAR*γ* agonists that inactivate cytoplasmic NF-*κ*B or ATRA that appears to decrease nuclear NF-*κ*B levels leads to decreased rotavirus infection in terms of decreased percentage of infected cells, viral antigen accumulated, and infectious titer. However, fatty acids are able to interfere with the inflammatory cell response via pathways other than those mediated by NF-*κ*B [28].

Virus-induced oxidative stress is a well-documented fact, particularly in the case of RNA viruses [34]. ROS production is correlated with cell death but also may play an important role by signaling the regulation of viral replication [34]. Rotavirus infection has been shown to induce ROS [35], whereas NAC treatment has been reported as an inhibitor of rotavirus infection both* in vitro* and* in vivo* [3, 5, 6]. In the present study, we found a relatively increased proportion of villus cells being positive to ROS assay following ECwt infection both* in vivo* and* in vitro*. However, PGZ treatment of infected villi led to a reduced mean percentage of ROS-positive villus cells that was very close to that observed in noninfected villus cells. The present findings support the hypothesis that PGZ inhibits the ECwt-induced oxidative stress in small intestine villus cells, interfering with viral infection.

Overall, our results indicate that rotavirus infection is able to increase the accumulation of cellular proteins PPAR*γ*, NF-*κ*B, PDI, and Hsc70 and ROS both* in vivo* and* in vitro* conditions, whereas PGZ and ATRA treatment returned these proteins to their basal levels and concomitantly reduced rotavirus infection. We propose that rotavirus infection is sensitive to the expression of genes regulated by transcription factors binding to PPREs and RAREs.

## 5. Conclusion

In conclusion, the results reported here provide evidence that PPAR*γ* agonists and ATRA exert an inhibitory effect on mouse intestinal villi infected* in vivo* with ECwt, causing a significant reduction of infectious virions and decreasing some of the cellular proteins involved in rotavirus infection. The effects of PPAR*γ* agonists and ATRA on rotavirus infection in a synchronous system consisting of small intestinal villi isolated from mice suggest that genes controlled by PPREs and RAREs are involved in the viral cycle inhibition. The PGZ inhibitory effect through PPAR*γ* activation gives us a better insight into the host cell response to rotavirus infection and the mechanisms involved in its pathogenesis. However the precise mechanisms of how PPAR*γ* agonist- and ATRA-induced genes regulate rotavirus infection cycle are not completely understood and deserve further research. Since PGZ has been withdrawn as therapeutic agent in some countries, other TZD could be potential candidates for rotavirus infection treatment. However, developing of therapeutic agents for rotavirus infection is dependent on better understanding of the cellular and molecular mechanisms underlying rotavirus infection.

## Figures and Tables

**Table 1:** Flow cytometric measurement of viral structural proteins and cellular proteins.

		SP-positive cells (%)	PPAR*γ*-positive cells (%)	NF-*κ*B-positive cells (%)	NF-*κ*B-p-positive cells (%)	COX-2-positive cells (%)	PDI-positive cells (%)	Hsc70-positive cells (%)
Uninfected mice^*∗*^		5.8 ± 1.08	0.2 ± 0.2	0.2 ± 0.2	0.2 ± 0.2	0.8 ± 0.4	2.6 ± 0.72	0.1 ± 0.14
ECwt-infected mice^*∗*^		23.6 ± 2.17	25.6 ± 2.26	18.9 ± 1.94	27.2 ± 2.33	37.4 ± 2.73	13.5 ± 1.64	7.7 ± 1.24
ECwt-infected mice + PGZ	12 h.p.i.	30.7 ± 2.48	20.3 ± 2.01	13.1 ± 1.62	13.1 ± 1.62	22.5 ± 2.12	8.3 ± 1.29	7.5 ± 1.22
	24 h.p.i.	21.0 ± 2.05	13.0 ± 1.61	14.1 ± 1.68	14.1 ± 1.68	20.3 ± 2.01	7.5 ± 1.22	6.7 ± 1.16
	36 h.p.i.	7.5 ± 1.22	28.3 ± 2.38	24.5 ± 2.21	27.4 ± 2.34	31.1 ± 2.49	19.7 ± 1.98	17.8 ± 1.89
	48 h.p.i.	0.1 ± 0.14	6.6 ± 1.14	6.8 ± 1.17	6.0 ± 1.1	8.2 ± 1.28	3.3 ± 0.81	2.6 ± 0.72

^*∗*^Mean percentage through 12–48 h.p.i. period.
